# Photopolymerization Pattern of New Methacrylate Cellulose Acetate Derivatives

**DOI:** 10.3390/polym16040560

**Published:** 2024-02-19

**Authors:** Ioana-Sabina Trifan, Andreea L. Chibac-Scutaru, Violeta Melinte, Sergiu Coseri

**Affiliations:** Polyaddition and Photochemistry Department, Petru Poni Institute of Macromolecular Chemistry, 41 A Gr. Ghica Voda Alley, 700487 Iasi, Romania; trifan.sabina@icmpp.ro (I.-S.T.); andreea.chibac@icmpp.ro (A.L.C.-S.)

**Keywords:** cellulose acetate, functional derivatives, functionalization reaction, photopolymerization, light irradiation, photocrosslinked networks

## Abstract

Polymeric photocrosslinked networks, of particular interest in the design of materials with targeted characteristics, can be easily prepared by grafting light-sensitive moieties, such as methacrylates, on polymeric chains and, after photochemical reactions, provide materials with multiple applications via photopolymerization. In this work, photopolymerizable urethane–methacrylate sequences were attached to free hydroxyl units of cellulose acetate chains in various proportions (functionalization degree from 5 to 100%) to study the properties of the resulting macromolecules and the influence of the cellulosic material structure on the double bond conversion degree. Additionally, to manipulate the properties of the photocured systems, the methacrylate-functionalized cellulose acetate derivatives were mixed with low molecular weight dimethacrylate derivatives (containing castor oil and polypropylene glycol flexible chains), and the influence of UV-curable composition on the photopolymerization parameters being studied. The achieved data reveal that the addition of dimethacrylate comonomers augmented the polymerization rates and conversion degrees, leading to polymer networks with various microstructures.

## 1. Introduction

The most abundant polysaccharide in the natural environment coming from renewable and low cost sources, cellulose is extensively used in industry and academic research owing to its exceptional properties, such as biocompatibility, total biodegradability, crystallinity, and lack of toxicity [1,2]. Unfortunately, the increased number of hydrogen bonds between the hydroxyl groups of the polymeric chains renders cellulose insoluble in water and most organic solvents, which has proven to be a severe nuisance in its immediate deployment on a much larger scale. Consequently, scientists employ cellulose derivatives, mostly generated by chemical oxidation methods [3,4,5], etherification [6,7], or esterification [8,9], that have good solubility in both water and common organic solvents. One functional cellulose derivative produced via cellulose alkylation, cellulose acetate (CA), for example, has been thoroughly investigated for use as a cellulose alternative in a variety of applications [10,11,12,13], exhibiting high film-forming capacity due to its excellent solubility in many organic solvents, in addition to the conventional features of cellulosic materials (biocompatibility, biodegradability, and low cost). Because there are less accessible hydroxyl groups, the hydrogen bond networks in CA polymeric chains are significantly diminished, resulting in increased flexibility. However, CA functionalization is still required to expand the range of currently available cellulose chemicals and identify appropriate uses [14,15,16,17]. One common alternative for functionalizing CA and polysaccharides, in general, is to employ molecules of low molecular weight from the (meth)acrylate derivative family that includes photopolymerizable fragments [18,19,20,21], taking into account that the recently generated products take part in photopolymerization processes and are susceptible to light irradiation. Using photochemical techniques rather than physical or chemical ones has a number of benefits, such as greater control over the entire process due to the ability to turn on and off the irradiation source and the ability to keep solvents out of the reactions [22,23,24].

Polysaccharides must have functional groups in their structure capable of polymerizing when exposed to light to be used in photopolymerization processes. This reaction also necessitates the presence of a photoinitiator, which can split and create new reactive species that could start the photopolymerization process, as well as the exposure of the sample to electromagnetic radiation from a source [23,25]. The resulting products are three-dimensional photocrosslinked networks remarkable for their unique properties and multiple applications in everyday life, e.g., as coatings, adhesives, or 3D printed objects [26,27,28]. Several variables affect the properties of the photocrosslinked samples. Among these are the sample’s nature and molecular weight functionalized with (meth)acrylate sequences, as well as the functionalization degree (FD), which affect the rate of photopolymerization, the conversion of double bonds, and, most importantly, the properties of the crosslinked network [29,30]. Therefore, a recent and significant area of macromolecular study is the thorough examination of the impact of FD on the characteristics of certain photopolymerized networks. FTIR spectroscopy is a straightforward and adaptable technique for tracking the photopolymerization of (meth)acrylic bonds in response to UV or visible light. It evaluates the changes in photopolymerizable bonds over time and, via kinetic studies, enables the calculation of the rate of photopolymerization as well as the degree of conversion as a function of time [31,32,33].

To achieve a range of photopolymerizable CA derivatives (CA-Mx) with functionalization degrees ranging from 5 to 100%, the current study reports an in-depth investigation into the functionalization of cellulose acetate with varying amounts of 2-isocyanatoethyl methacrylate (2-IEMA). The chemical structures of modified-CA were confirmed by spectral studies (FTIR and ^1^H NMR), whereas the changes in chemical, physical, and thermal characteristics were examined by specific analyses (XRD, TGA, and contact angle), while the photopolymerization profile of the urethane–methacrylate functionalized CA was investigated by FTIR spectroscopy. Considering the high molecular weight (Mn~50,000 g/mol) of the cellulose acetate, control of the photocrosslinking process can be a challenging task. To address this, the photopolymerization behavior of CA derivatives was enhanced by the introduction of certain low molecular weight dimethacrylate derivatives (containing flexible polypropylene glycol chains and castor oil). This method generated higher crosslinking conversions and a more porous morphology. Thus, by ingeniously producing these cellulosic derivatives, we can modify their physical characteristics and successfully employ these new compounds in the development of novel cellulose-derived materials that exhibit greater solubility in common organic solvents.

## 2. Experimental Section

### 2.1. Materials

Cellulose acetate (Mn~50,000 g/mol by GPC, 39.7 wt% acetylation degree), anhydrous tetrahydrofuran (THF), dibutyltin dilaurate (DBTDL), 2-isocyanatoethyl methacrylate (2-IEMA) with 98% purity, and Irgacure 819 photoinitiator, were purchased from Sigma-Aldrich Chemical Co. Wyoming, IL, USA, and used as they were received, without any purification in advance. The synthesis and characterization of castor oil urethane methacrylate (CO-UDMA) and the methacrylate derived from polypropylene glycol (PPG-M) were described in a previously reported study [34].

### 2.2. Synthesis of Cellulose Acetate Derivatives (CA-M5–CA-M100)

The grafting of 2-IEMA to the available hydroxyl groups of the anhydroglucose units of CA allowed the attachment of methacrylate moieties onto CA via urethane-type linkages. In order to achieve different degrees of functionalization, the composition of the reaction mixtures was modified according to the data presented in Table 1, so that the CA-Mx series was obtained, where x represents the corresponding FD (%) for each product as follows: CA-M5, CA-M10, CA-M25, CA-M50, and CA-M100 corresponding to a functionalization degree of 5, 10, 25, 50, and 100%, respectively.

All functionalization reactions proceed in the same manner and consist of the dropwise addition of 2-IEMA diluted in THF into the homogeneous solution of CA dissolved in THF, which occurred in the presence of catalytic amounts of dibutyltin dilaurate (0.05 wt% with respect to 2-IEMA). The reactions were stirred at 40 °C for 24 h and monitored by recording FTIR absorbance spectra from which the isocyanate stretching bands at 2260 cm^−1^ were shown to diminish until they were completely absent. The CA-Mx compounds were obtained as white powders following solvent evaporation and cooling to room temperature.

### 2.3. Preparation of CA-Mx Photopolymerized Films

For each CA-Mx sample, a homogenous mixture composed of CA derivative and Irgacure 819 photoinitiator (1 wt%), solubilized in a small amount of THF, was obtained. All mixtures were cast as coatings on a Teflon plate and irradiated with an LA 500 Blue Light lamp, Eurolite, Waldbüttelbrunn, Germany, in the visible domain up to 300 s, which led to the formation of CA-derivative-based crosslinked networks as photopolymerized films.

The photopolymerized films based on CA-Mx samples in a mixture with PPG-M or CO-UDMA were obtained by preparing homogenous mixtures composed of CA-M, Irgacure 819 photoinitiator (1 wt%), and PPG-M or CO-UMDA, solubilized in THF and cast as coatings on Teflon plates. The irradiation was performed following the protocol described for the CA-M films formation.

### 2.4. Characterization

Using the potassium bromide (KBr) pellet method, FTIR experiments for NCO band tracking and structural identification were carried out on a Shimadzu IRAffinity-1S Spectrophotometer, Shimadzu, Kyoto, Japan. Each transmittance spectra with its unique bands occurring in the 4000–400 cm^−1^ region was obtained after a total of 32 scans. Using deuterated dimethyl sulfoxide to solubilize the compounds, the Bruker Avance Neo 400 spectrometer, Bruker, Billerica, MA, USA, was used to record the ^1^H and ^13^C NMR spectra in order to clarify the chemical structure of the CA and CA-Mx products. For X-ray diffraction (XRD) analysis, a Rigaku Miniflex 600 X-ray diffractometer, Rigaku, Tokyo, Japan, as used to record the XRD patterns in the range of 2θ = 4.0°–50.0°, applying Cu Kα radiation (λ = 1.5406 Å), with the step size of 0.0025° and recording rate of 2° per minute. Water contact-angle measurements were performed on a KSV CAM 101 goniometer (KSV Instruments Ltd., Helsinki, Finland) at room temperature by placing at least six droplets of double-distilled water with a volume of ~1 μL on different spots of each sample coated as plane films. The contact angles for all films were automatically calculated by fitting the droplet shape and were presented along with the corresponding standard deviation. For thermogravimetric analysis (TGA), about 3–12 mg of the solid samples were weighed and placed in alumina pans for TG measurements. Each sample was heated from 30 °C to 700 °C, with a heating rate of 10 °C min^−1^, under a dry nitrogen atmosphere at a flow rate of 50 mL min^−1^, using an STA 449F1 JUPITER (Netzsch, Selb, Germany) device. The data were processed with the NETZSCH PROTEUS 4.2 software.

The photopolymerization kinetics were evaluated by FTIR spectroscopy, using the KBr pellet method, and recording the absorbance spectra on an IRAffinity-1S spectrophotometer. To monitor the photopolymerization patterns, the photopolymerizable derivatives and Irgacure 819 (1 wt%) photoinitiator, diluted with small amounts of tetrahydrofuran, were thoroughly mixed to achieve a homogeneous composition. Further, the homogeneous compositions were cast in thin layers on KBr pellets and were exposed to UV irradiation at different times (2 s, 5 s, 15 s, 30 s, 60 s, 120 s, 200 s, and 300 s). The light source used for irradiating the samples was an LA 500 Blue-Light lamp (λ = 400–500 nm, light intensity = 2 mW cm^−2^).

The morphology of cross-sectioned, photopolymerized CA-Mx, CA-Mx–PPG-M, and CA-Mx–CO-UDMA films was investigated with SEM analysis using a Verios G4 UC (Thermo Fisher Scientific, Waltham, MA, USA) scanning electron microscope used in low vacuum mode at an accelerating voltage of 10 kV. To increase the films’ conductivity and resistance against high vacuum conditions, each sample was covered with a thin layer of platinum. The microphotographs of photopolymerized films in the cross-fractured section were registered with a magnification of 5000×.

## 3. Results and Discussion

**Figure 1:**
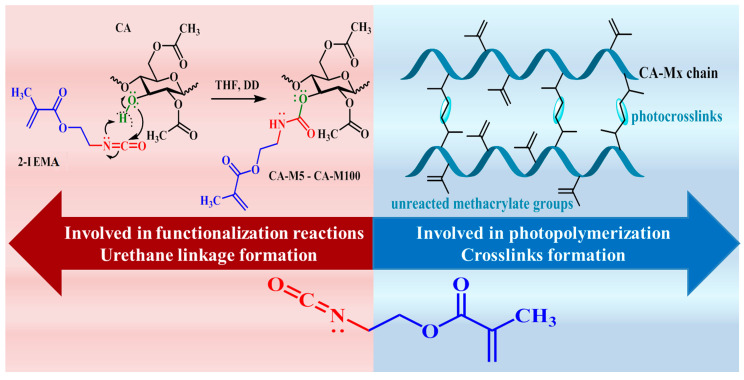
The two reaction routes involving the attachment of 2-IEMA emphasising the corresponding products formation.

Two sequences define the structure of 2-IEMA, both being involved in different types of reactions: addition and photopolymerization. The addition reaction consisted of a nucleophile attack of the nitrogen electron pair on the hydrogen from the hydroxyl group, followed by structural rearrangements and a new C-O simple bond formation as a part of the urethane unit, while the photopolymerization of the methacrylate units led the formation of photocrosslinks between the polymer chains, Figure 1. The following content emphasizes the basic properties of the CA-Mx properties and focuses on their photochemical activity when used alone or in combination with other light-sensitive compounds.

### 3.1. Investigation of the Chemical Structure

**Figure 2:**
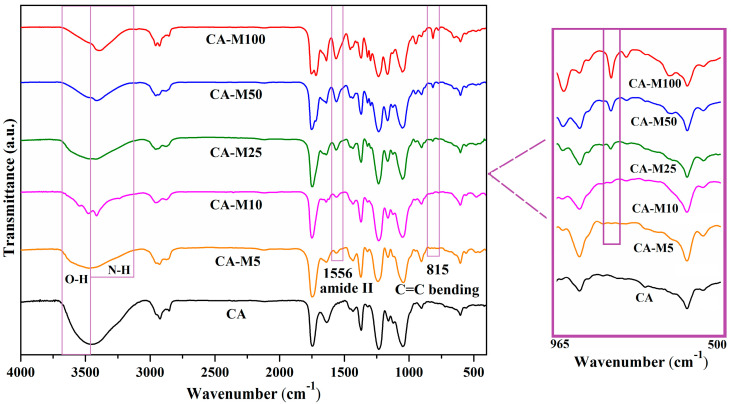
Possible reaction pathways for the structural sequences of 2-IEMA and the formation of the corresponding products.

The properties of the CA-Mx samples were investigated by using various analysis techniques, including FTIR and NMR spectroscopies for chemical structure identification, TGA analysis for acknowledgment of their thermal stability, and contact-angle measurements, in order to determine their hydrophilic/hydrophobic behavior. For the commercial CA, the FTIR transmittance spectrum highlights absorption bands at 3447 cm^−1^, which corresponds to the hydroxyl group (O-H) stretching vibration, and at 1744 cm^−1^, which is characteristic of C=O stretching from the ester group (-O-CO-CH_3_), but does not present any band at 815 cm^−1^, nor at 1638 cm^−1^. Instead, spectra of the modified CA samples contain bands at both of these wavenumber values that correspond to the carbon–carbon double bond (-C=C-) from the grafted methacrylate units, bending at 815 cm^−1^, and stretching at 1638 cm^−1^. Compared to the CA spectrum, in the spectra of CA-Mx samples, the specific band for the hydroxyl group at 3447 cm^−1^ undergoes a decrease as related to the reduced number of hydroxyl groups in CA derivatives than in the CA [11,35]. Moreover, in the spectra of the derivatives, the bands characteristic to the new-formed urethane linkages appeared at 3300 cm^−1^, 1640 cm^−1^, and 1556 cm^−1^, which are attributed to the N-H stretching and amide bending vibrations but may shift and overlap with other specific bands such as the ones of methacrylate or hydroxyl groups (Figure 2). These aspects indicate that the CA functionalization reactions were indeed accomplished [11,36].

**Figure 3:**
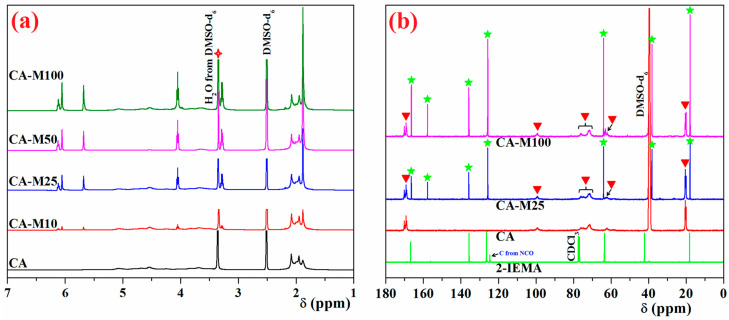
^1^H NMR (**a**) and ^13^C NMR (**b**) spectra of initial (CA and 2-IEMA) and functionalized CA-Mx derivatives. The red asterisk, green asterisk, and red triangle highlights the structural changes that occur after the reaction, highlighting the change in the intensity of the ^13^C-NMR signals for the carbon atoms.

As it is about to be shown below, in Figure 3a, the appearance of new peaks in the ^1^H NMR spectra of functionalized CA samples indicates the chemical changes suffered by CA during functionalization. In the CA spectrum, the protons corresponding to the anhydroglucose unit appear between 3.6–5.1 ppm, in the aliphatic region. In addition to these protons, the CA-Mx samples’ spectra highlight protons belonging to the methacrylate and urethane sequences attached to the hydroxyl groups of the basic structure. The methyl protons attached to the double bond present the most increased shielding, their peaks appearing at 1.9 ppm; instead, the protons from the methylene attached to the urethane and ester linkages are located at 3.3 and 4.05 ppm. As for protons of the double bond, they distinguish from each other and have two peaks, at 5.6 and 6.1 ppm [37]. Even more, from the ^1^H NMR spectra, the FD (%) of each CA-Mx compound was calculated from the integral ratio of the newly arising unsaturated protons, appearing at 5.6 and 6.1 ppm to that of the protons belonging to the anhydroglucose repeating unit, which appear in the region of 3.6–5.1 ppm. As assumed, the FDs (%) were appropriate to the theoretical FDs (%) given the high reactivity of isocyanate sequences in the IEMA derivative [11].

For a more thorough investigation, the ^13^C NMR spectra for the initial materials (2-IEMA and CA) and for two selected functionalized samples were recorded, and the results are graphically represented in Figure 3b. As illustrated in the figure, the main peaks attributed to the carbon atoms from the anhydroglucose units are located at 99 ppm, 71.5–76 ppm, 62.3 ppm, and 20.2 ppm, with the carbonyl atoms (C=O) from acetate at 169 ppm, while the carbon atoms corresponding to the urethane–methacrylate sequences give the signals at 166.5 ppm (C=O), 157.8 ppm (O-CO-NH-), 135.5 and 125.8 ppm (CH_2_=C-), 64 ppm (-CO-O-CH_2_-), 38 ppm (-CH_2_-NH-), and 18 ppm (-CH_3_), confirming the attachment of methacrylic units to the cellulosic chains.

Interpretations of the ^1^H NMR, ^13^C NMR, and FTIR spectra revealed that CA derivatives had varying FDs (%) between 10 and 100%, indicating that CA was effectively functionalized with methacrylic moieties through urethane linkages.

### 3.2. X-ray Diffraction (XRD) Analysis

**Figure 4:**
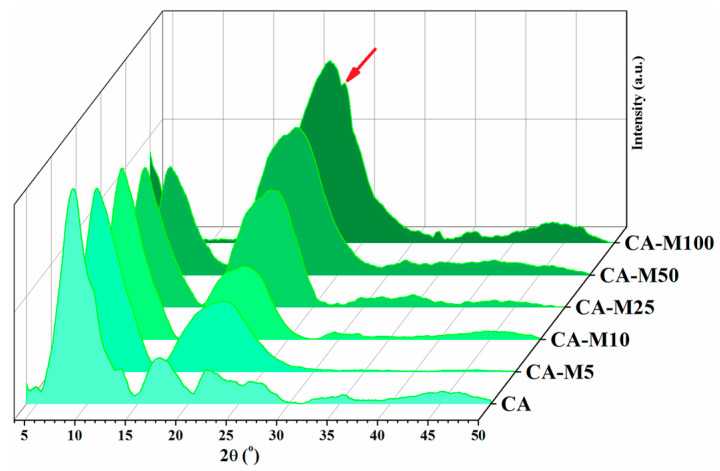
XRD patterns of CA and modified CA samples showing the existing (semi)crystalline and amorphous regions in each compound. The red arrow indicates the appearance of the new crystalline peak located at 23.2°.

The crystallinity modification of various compounds can be easily evaluated by investigating the nature of Bragg’s peaks from the XRD patterns. It is a well-known issue that thermal history has a substantial impact on the crystallization processes of different types of materials, and cellulose derivatives are no exception [38,39,40]. The frequently used method to achieve samples with a similar thermal background is thermal annealing; however, this technique is not suitable in the case of our samples substituted with various amounts of double bonds that are susceptible to thermal polymerization [41]. Therefore, the specimens for XRD investigation were cast from THF solutions poured into glass plates and evaporated into the air under a controlled temperature (23 ± 1 °C) for 72 h. The XRD pattern of the CA sample exhibits a sharp peak at 8.6°, which is associated with the semicrystalline acetylated cellulose [42], accompanied by some low intensity peaks at around 2θ = 13.2°, 17.2°, and 22°, correlated with the reduced crystallinity of cellulose acetate sample [43,44]. After functionalization, the diffraction peak at 8.6° manifests a proportional tendency to decrease in the spectra of CA derivatives as the FD (%) increases until it completely vanishes for the CA-M100 sample, as its structure was modified the most. Upon modification, in the XRD diffraction patterns of the CA-Mx samples, the formation of a new broad peak centered at around 21–21.5° is visible, which is assignable to the development of an amorphous structure of cellulosic samples that is proportionally increasing with the modification degree [45]. Also, it is possible that the poor crystallization outcomes can be attributed to the samples’ preparation by solvent casting [46] without thermal annealing at elevated temperatures. However, a mixed amorphous crystalline phase could be assumed in the case of the CA-MA100 sample, for which the shoulder at about 23.2° can be ascribed to the formation of newly ordered structures associated with a crystallization pattern triggered by methacrylic sequences (Figure 4) [21,35,45].

### 3.3. Investigation of the Wettability of Cellulose Acetate and Its Derivatives

As with any other chemical compound, polysaccharide derivatives show specific behavior towards the liquids, including water. Based on the value of the contact angle formed between the sample’s surface and a water droplet, the compound can be either hydrophilic or hydrophobic; the reference value being 90°. Compounds that form with water at an angle under this value have an affinity for it, while the contact over 90° suggests their hydrophobic behavior. In order to determine the contact angle, a polymeric plane film must be formed on which a few water droplets are added. The final value of the contact angle is the average number of each droplet’s contact angle [47].

CA and CA derivatives were dissolved in THF in six vials, stirred on a magnetic hob, and coated as films on which six droplets of water were added per film. The measured angle value for the CA was 62 ± 1° which was very close to the value described in the scientific literature (~60°) for the pure CA membranes (M = 50,000 g/mol having 29–45% acetyl group) [48,49]. Modification in the angle values may refer to the fact that 2-IEMA reacted with CA, affecting the existing hydrogen bonds between polymeric chains, either by decreasing their number or by increasing their length. Both cases lead to the formation of CA derivatives with a more pronounced hydrophilic behavior. In Table 2, the statistical water contact angles of the CA and CA-Mx samples are listed.

Although the FD (%) influences the properties of the CA-Mx samples, including the affinity towards the water, which should be slightly pronounced for each in comparison to CA, several other factors—the molecular mass and its distribution, the density of grafted functional groups, heterogeneous functionalization, intermolecular interactions, roughness of the film surface and film porosity—interfere and produce changes of the water contact-angle values [50]. The water contact angles of the CA-M5 and CA-M50 films are larger than those of the CA film, suggesting that these derivatives behave with less hydrophilic behavior, possibly as a result of the heterogeneous functionalization which leads to an increase in the roughness of the film surface [51]. In the case of the CA-M100 film with the lowest value of the contact angle, the increase in hydrophilicity may be attributed to a more homogenous functionalization and, implicitly, to the smoother surface of the film, and also to the increase in the surface energy of the material due to the higher number of carbonyl (-C=O) groups present in the CA-M100 derivative and their intramolecular interaction with the other functional groups of the material [50,52]. A smoother surface and higher surface energy promote better wetting and lower the contact angle [52,53]. Due to these reasons, the statistic contact angles do not respect a specific pattern. Regardless of this aspect, all compounds had water contact-angle values below 90°, suggesting their hydrophilic interactions with the water molecules [54].

### 3.4. Thermal Stability Investigation

**Figure 5:**
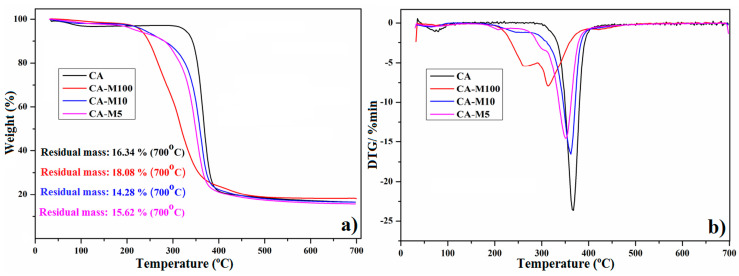
TG curves (**a**) and DTG curves (**b**) of CA and methacrylated CA derivatives.

TGA is an efficient technique to investigate the surfaces and inner layers of polymeric compounds. It implies the samples’ degradation by constantly increasing the temperature and investigation of the possible transformations occurring during this process. Through this characterization technique, we confirmed the attachment of the 2-IEMA sequences on CA and determined the thermal stability of CA and CA-M5–CA-M100. Also, we were able to investigate whether the FD (%) has influences on these properties. Figure 5a shows the weight-loss (TG) curves for CA, CA-M5, CA-M10, and CA-M100 products, which indicate different stages in the decomposition during the temperature increase. It appears that CA exhibits good thermal properties due to its high glass-transition temperature (T_g_) [12,55]. Low degradation temperatures, as low as 70 °C, cause the leftover solvent to evaporate or turn to water (molecules weakly bonded, adsorbed onto the film surface or which fill the pores), as CA and its derivatives are hydrophilic compounds that can easily capture water through the hydrogen bonds formed with hydroxyl groups [55,56]. This process continues with the breakdown of the ester groups, the outer layers, the breakage of glycoside units, and linkages that comprise the CA skeleton and terminate before 400 °C. It was observed that the thermal stability of CA is greater than that of its derivatives. These results may be attributed to the grafting of novel functional groups into the cellulose acetate chain. These functional groups are more vulnerable to thermal degradation at higher temperatures, resulting in a decrease in thermal stability [57,58], and the CA-M100 sample with a higher content of urethane–methacrylic units are more susceptible to thermal decomposition. Moreover, the high number of functional groups of CA-M100 can restrict the mobility of polymer chains, with the derivative becoming more rigid and less able to dissipate heat, and, thus, resulting in a loss in thermal stability.

Nevertheless, the thermal decomposition of CA derivatives starts above 200 °C, a satisfactory temperature for the envisaged applicability field. The weight-loss percentages at 700 °C were determined from the DTG curves, and, for all derivatives, including CA, varied between 82 and 86%, as already reported for cellulose acetate and its derivatives [55,59].

### 3.5. Photopolymerization Kinetics of Cellulose Acetate Derivatives (CA-M5–CA-M100)

Given the fact that CA was modified with methacrylate moieties, the photobehavior of CA-M samples was studied by investigating their response to light irradiation after different irradiation intervals.

#### 3.5.1. Investigation of the Photopolymerization Kinetics of CA-Mx Samples

**Figure 6:**
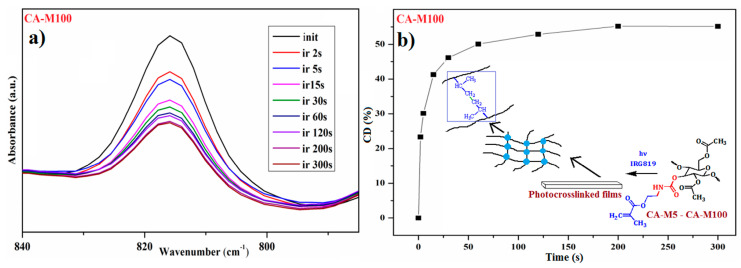
FTIR absorbance spectra (**a**) and the conversion degree (**b**) of photopolymerizable C=C bonds in a CA-M100 sample monitored at 815 cm^−1^ during various irradiation stages.

The evolution of the CA-M samples’ photopolymerization reactions was evaluated by using FTIR spectroscopy for kinetic purposes. The FTIR absorbance spectra, measured as a function of irradiation time, highlight the decreasing of the photopolymerizable carbon–carbon double bond characteristic bands at 815 cm^−1^ and 1638 cm^−1^. But, given the fact that the band at 1638 cm^−1^, which corresponds to the stretching vibration, overlaps with the band for amide I, it was a better option to focus only on the decreasing of the band at 815 cm^−1^. Figure 6a represents the FTIR spectrum of the CA-M100 sample, which highlights the differences between the intensity of the double bond bands at different irradiation times. Additionally, the conversion degrees were calculated from the FTIR absorbance spectrum with the formula [37]:

(1) $$\mathrm{CD}(\%)=(1-\frac{At}{A0})\times 100$$

where *At* is the absorbance for the methacrylate group band at 815 cm^−1^ in the derivatives spectra after a given irradiation time, and *A*0 is the absorbance observed in the spectra before irradiation.

**Figure 7:**
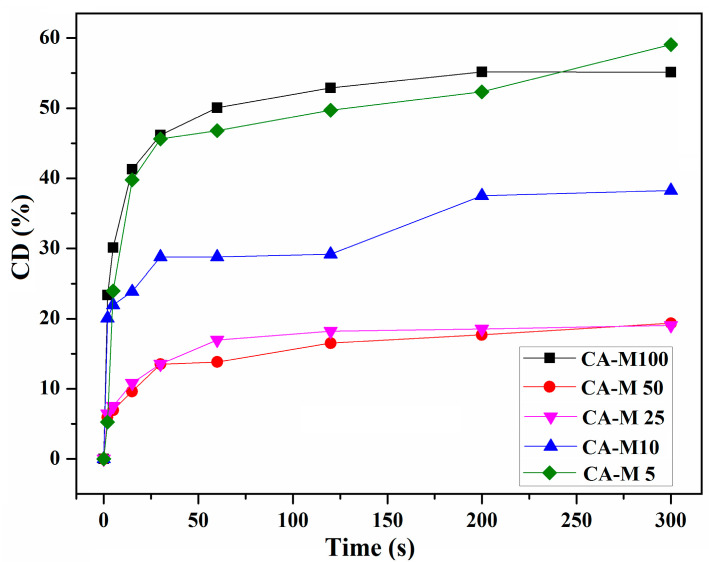
CD (%) of the CA-Mx series (CA-M5–CA-M100).

The curves attributed to the conversion degrees (CDs%) of the CA-Mx samples after predetermined irradiation times are presented in Figure 7. The maximum value of CD (%) after 300 s of irradiation, which corresponded to the CA-M5 sample, was 59.06%, followed by 55.14% for the CA-M100 sample. During the photocuring process, the CDs (%) and photopolymerization rates are strongly influenced by the volume shrinkage which occurs by distance reduction between the functional groups.

Given the fact that the CDs (%) were moderate and no major differences appeared in the FTIR spectra for the methacrylate absorption bands at 815 cm^−1^ during irradiation, it was assumed to contribute to the method optimization by combining CA samples with chemical compounds characterized by greater photoreactivity. Therefore, the crucial task relied on finding suitable compounds that could be used in combination with CA derivatives for the photopolymerization process improvement by increasing the CDs (%) values.

#### 3.5.2. Photopolymerization Kinetics of CA-Mx Samples Used in Combination with Low Molecular Weight Photopolymerizable Monomers

**Figure 8:**
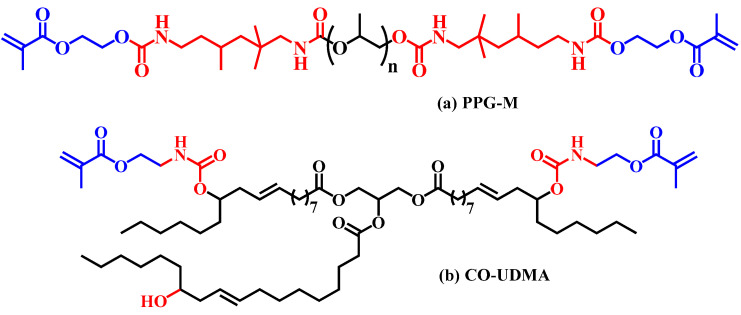
Chemical structures of PPG-M and CO-UDMA used in combination with CA-Mx samples for improvement of the photopolymerization process.

Polysaccharides, such as cellulose and its derivatives, are polymers that are well known for their high molecular mass that has a great influence on the derivatives FD (%) and also represent a crucial factor in forming crosslinked networks via photopolymerization. One method to improve the crosslinking density of CA-Mx-based photocrosslinked networks is relying on finding other macromolecular compounds that could be mixed up with CA-Mx samples. By combining methacrylate CA derivatives bearing photopolymerizable sequences and low molecular mass compounds, the crosslinking density is increased, which also implies higher values for CDs (%) of the C=C double bond from the methacrylate sequences. Considering their chemical structure, as well as their relatively low molecular mass and the ability to form transparent films, urethane methacrylate derived from polypropylene glycol (Figure 8a) and modified castor oil with urethane-methacrylate units (Figure 8b) were chosen to be combined with CA-Mx samples in order to obtain light-induced crosslinked networks with an enhanced crosslinking degree.

In Table 3 are presented the gravimetric ratios of the components for the proposed formulations containing both the methacrylate derivative of cellulose acetate and the urethane dimethacrylate comonomers.

#### 3.5.3. Investigation of the Photopolymerization Kinetics of CA-M Samples in a Mixture with PPG-M or CO-UDMA

**Figure 9:**
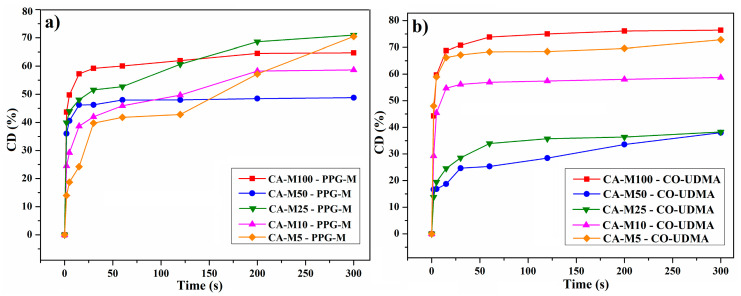
CD (%) of the CA-Mx–PPG-M films (**a**) and of the CA-Mx–CO-UDMA films (**b**).

The FTIR absorbance spectra were recorded as a function of the irradiation times using FTIR spectroscopy. The decreasing of the bands corresponding to the photopolymerizable double bonds was monitored from the spectra at 815 cm^−1^ for both CA-M100–PPG-M and CA-M100–CO-UDMA films. The CDs (%) for CA-Mx–PPG-M and CA-Mx–CO-UDMA films were calculated from each FTIR spectrum with the same formula as previously used and included in Figure 9.

**Figure 10:**
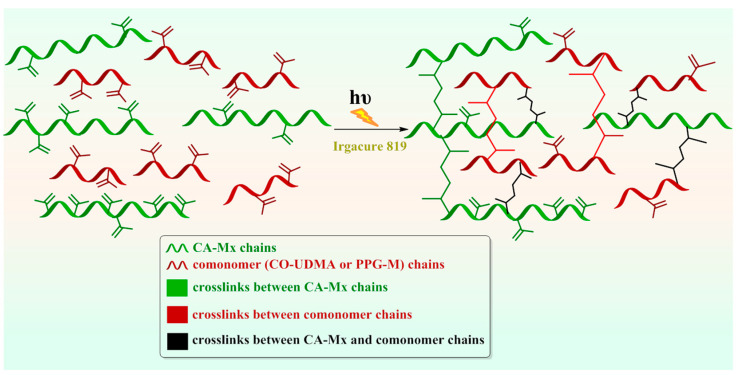
Schematic idealized representation of the photopolymerization process with the formation of a crosslinked network.

According to the data illustrated in Figure 9, both compounds added in a mixture with CA derivatives made a slight improvement to the CDs (%) of the photopolymerizable sequences at each irradiation time, as well as to the crosslinking degree of the polymeric networks. To illustrate the possible crosslinking mechanism between the cellulose acetate urethane methacrylates and the dimethacrylate macromonomers, to highlight the possible structural modifications, and also to justify the significant variance of double bond conversion degree, an idealized graphical representation of the possible interactions that occur during photochemical irradiation is given in Figure 10.

**Figure 11:**
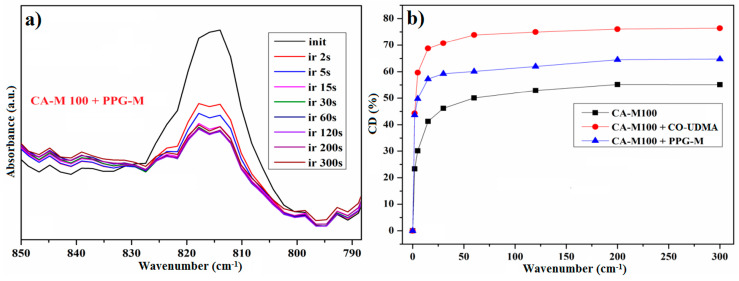
FTIR spectra of the CA-M100–PPG-M film (**a**) and comparison between the CDs (%) of CA-M100, CA-M100–PPG-M, and CA-M100–CO-UDMA films (**b**).

Figure 11a represents the FTIR spectra of CA-M100–PPG-M during irradiation from where the evolution of the double bond band was monitored, while Figure 11b indicates a comparison between the CDs (%) after the irradiation time, corresponding to the three types of CA-M100-based polymeric films.

**Figure 12:**
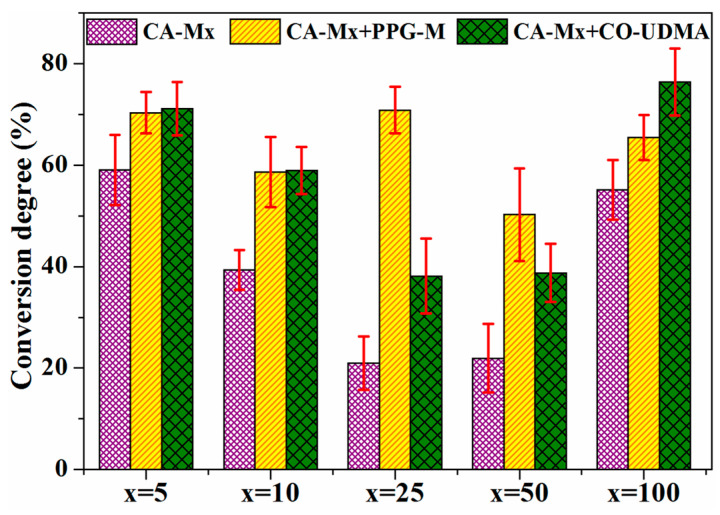
Conversion degree of CA-M5–CA-M100, alone and in combination with PPG-M or CO-UDMA, after an irradiation time of 300 s.

Consequently, Figure 12 symbolizes a schematic representation of the CDs (%) reached after 300 s of irradiation, corresponding to each CA derivative used alone or mixed with PPG-M or CO-UDMA. Unlike the rest of the CA derivatives-based films, films based on CA-M10 have similar CDs (%), around 59%, whether they contain PPG-M or CO-UDMA as the second compound of the mixture. Out of the films based on CA derivatives and modified castor oil, CO-UDMA and CA-M100-containing film have the maximum value of the CD (%), which is around 76%. As for the PPG-M-based films, CA-M25 –PPG-M has the highest value of the conversion degree, around 71%. Moreover, the CDs (%) seemed smaller for the films having CA derivatives with intermediate values of FD (%) than CDs (%) of films containing CA-M5 derivative. The intricate process of photopolymerization is subject to various influences, including but not limited to viscosity, the presence of hydrogen bonds, the number and arrangement of photopolymerazable methacylic groups, and the microenvironment [60,61]. Hence, the higher photoreactivity of CA-M100 is most probably due to the existence of an increased number of photopolymerizable groups per CA chain. Meanwhile, the higher photoreactivity of CA-M5 may be attributed to a good mobility of polymer chains which ensure a sufficient availability of methacrylic units during photopolymerization. The lower photopolymerization degree achieved for the other CA derivatives with intermediate degrees of functionalization (10%, 25%, and 50%) can be explained by the diffusion restrictions that appeared due to the crosslinking that limits the diffusion of reactive species, and implicitly their accessibility for polymerization, as well as the existence of steric hindrance [60,61,62]. However, as can be observed from Figure 12, the photoreactivity of CA-Mx derivatives can be improved by combining them with one of the oligomers PPG-M or CO-UDMA, which will produce networks with the appropriate double bound transformation and crosslinking density for the intended usage.

#### 3.5.4. Morphological Characterization of Photopolymerized CA Derivatives-Based Films

**Figure 13:**
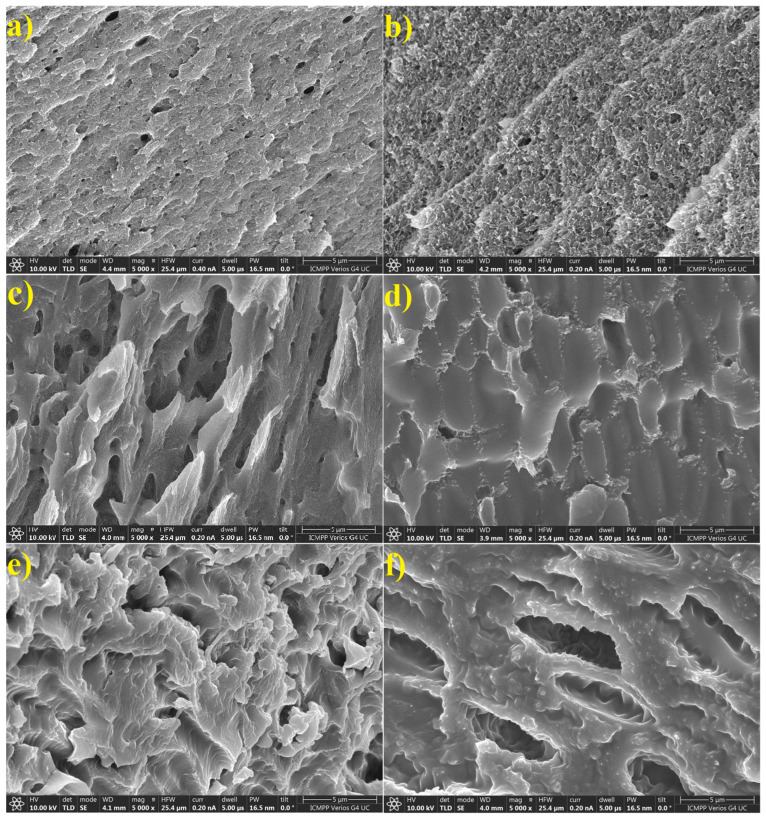
SEM microphotographs (5000× magnification) of CA-M50 (**a**), CA-M10 (**b**), CA-M50–PPG-M (**c**), CA-M10–PPG-M (**d**), CA-M50–CO-UDMA (**e**), and CA-M10–CO-UDMA (**f**).

To avoid possible damage to the photocrosslinked network, first, the films were frozen in liquid nitrogen, and then cross-fractured for morphology investigations. Figure 13 displays SEM microphotographs of the photopolymerized compounds, realized in cross section.

As for films based only on CA-M50 and CA-M10, the morphological studies indicate homogenous and compact regions, with few little pores appearing, most probably as a fracture consequence or because of the used solvent. Cross sections of photopolymerized films with two compounds combined present an increased porosity, leading to the formation of macrovoids that may seem to respect an organized pattern, especially for the composed film CA-M10–CO-UDMA. Films combining CO-UDMA and CA derivatives are materials characterized by a uniform organization, as well as a better pore size distribution, compared to films containing modified CA and comonomer PPG-M, which are more porogen, with interconnected large pores. Average values of the pore diameters were determined to be 0.9–1.1 + 3 μm for the CA-M50 film, which are higher than the values obtained for the CA-M10 film (only −0.6–0.7 μm). The diameter of pores reaches between 1.7 and 1.8 μm for both composed films containing CA-M50 (CA-M50–PPG-M and CA-M50–CO-UDMA), although some other pores with diameters of 1.4–1.5 μm or over 2 μm had been noticed. As expected, films with an increase in acetyl groups (low FD (%)) have a better organization of pores [51]. Therefore, the composed films based on CA-M10 had pores with a diameter of 4.4–4.5 μm (CA-M10–CO-UDMA) and 7.4–7.5 μm (CA-M10–PPG-M).

## 4. Conclusions

To create photocrosslinked polymeric networks through light-induced polymerization reactions, methacrylate-modified CA samples with an FD (%) ranging from 5 to 100% were created. These samples have better qualities than unmodified CA, particularly greater solubility in organic solvents and photopolymerizable sequences. FTIR absorbance spectra showed the development of the photopolymerizable double bonds over time during the irradiation time frame, where the methacrylate characteristic bands diminished as the irradiation duration rose. The conversion showed an overall increasing trend as anticipated, and conversion degrees were determined for each sample. However, considering alternatives such as combining CA and CA-M samples with chemical compounds with higher photoreactivity due to their low molecular mass and numerous photopolymerizable units—as suggested by the moderate conversion degree values—is worthwhile. The scientific literature states that photocrosslinked networks based on polysaccharides that are exhibited as flexible films function as macromolecular matrices (composites) in which low molecular mass compounds and nanoparticles can be created in situ or encapsulated. We can confidently state that, after taking into account all that was covered in the earlier parts, the field of photopolymerization research necessitates in-depth investigation and meticulous characterization of the photopolymerizable materials and the applications that go in addition to them.

## Figures and Tables

**Table 1:** Amounts of raw materials used for the synthesis of CA-M5–CA-M100 derivatives.

Product Name	CA (g)	CA (mmol)	2-IEMA (mL)	2-IEMA (mmol)
CA-M5	5	0.1	0.14	1
CA-M10	5	0.1	0.29	2
CA-M25	5	0.1	0.72	5
CA-M50	5	0.1	1.44	10
CA-M100	5	0.1	2.88	20

**Table 2:** Statistic contact angles of CA and CA-M5–CA-M100 with water.

Sample	WCA
CA	62 ± 1°
CA-M5	75 ± 1°
CA-M10	65 ± 1°
CA-M25	57 ± 1°
CA-M50	78 ± 1°
CA-M100	44 ± 1°

**Table 3:** Gravimetric ratios of the photopolymerized film compositions.

Films	CA-Mx (wt%)	PPG-M (wt%)	CO-UDMA (wt%)	Irg 819 (wt%)
CA-Mx–PPG-M	50	50	-	1
CA-Mx–CO-UDMA	50	-	50	1

## Data Availability

Data are contained within the article.
